# Factors associated with career decision-making difficulties among undergraduate nursing students: a latent profile analysis

**DOI:** 10.3389/fmed.2025.1644508

**Published:** 2026-01-06

**Authors:** Hongting Zhou, Liebin Huang, Zhijian Fu, Lin Li, Zhenzhen Huang, Junru Zhang, Mengjie Tang, Xinan Wang, Weijin Sui, Yiyu Zhuang

**Affiliations:** 1 Department of Nursing, Zhejiang University School of Medicine Sir Run Run Shaw Hospital, Hangzhou, China; 2 Faculty of Public Health and Nursing, Hangzhou Normal University School, Hangzhou, China

**Keywords:** career decision-making difficulties, latent profile analysis, learning engagement, nursing students, perceived social support, undergraduate

## Abstract

**Background:**

Career decision-making difficulties are often associated with negative outcomes, including decreased motivation for learning, weakened professional identity, increased burnout, and a higher risk of turnover. However, most research has concentrated on the overall levels of career decision-making difficulties, overlooking the potential variations among different student subgroups. This gap hinders the creation of targeted intervention strategies.

**Purpose:**

This study aimed to identify latent profiles of Career decision-making difficulties among undergraduate nursing students and explore factors associated with these profiles.

**Design:**

A cross-sectional study.

**Methods:**

A convenience sampling method was employed to recruit 562 undergraduate nursing students from three universities in China. Participants completed validated measures assessing demographics, learning engagement, perceived social support, and career decision-making difficulties. Latent profile analysis identified distinct profiles of career decision-making difficulties, and multinomial logistic regression examined the associated factors.

**Results:**

Three distinct career decision-making difficulties profiles emerged: multidimensional decision-making block (58.5%), knowledge-action disconnection (34.0%), and information-driven advantage (7.5%). Perceived social support, learning engagement, and class leadership status were significantly associated with these career decision-making profiles.

**Conclusion:**

Nursing students face diverse career decision-making challenges, highlighting the need for tailored institutional support. Students in the “Multidimensional Decision-Making Block” profile may benefit from structured career development courses. For the “Knowledge-Action Disconnection” profile, strengthening career information processing is crucial. Enhancing learning engagement, decision-making skills, and support networks can reduce uncertainty and better prepare students for their professional futures.

## 1 Introduction

The global shortage of healthcare workers remains a significant challenge. According to the World Health Organization (WHO), the worldwide deficit of healthcare professionals could reach 12.9 million by 2035 (1). This issue is particularly evident in the nursing sector, where the rate of nurse attrition greatly exceeds the number of new entrants into the profession (2). Consequently, nurse shortages persist as a significant problem, even in developed countries such as the United States and Canada (3). By the end of 2022, China had more than 5.2 million registered nurses; however, this number still falls short of meeting the increasing demand for healthcare services (4). As the largest group within the healthcare workforce, nurses are essential to healthcare delivery. The ongoing shortage of nursing staff will have negative consequences for healthcare resource allocation, service quality, and the ability to respond effectively to public health emergencies (5).

To alleviate this issue, governments and relevant institutions must implement measures to enhance the attractiveness of nursing careers and encourage the replenishment of talent. While existing research has focused on enhancing nurses’ psychological resilience, promoting career development, and optimizing work environments to reduce turnover rates (6, 7), early interventions should focus on nursing students who are making career choices. As the nursing profession continues to evolve, the demand for high-quality nursing professionals remains strong (8). Undergraduate nursing students are a vital part of the future nursing workforce. Their career choices significantly impact the stability of the nursing workforce and the quality of services provided. If nursing students face difficulties during their career decision-making process, they may hesitate or even abandon the nursing profession altogether. Therefore, it is crucial to address the challenges that undergraduate nursing students encounter as they make decisions about their careers.

Career decision-making difficulties (CDMD) are challenges individuals experience when they are uncertain about which profession to select or must choose among several options at the concluding stage of making career decisions. These difficulties are typically categorized into three main areas: insufficient readiness, inadequate information, and conflicting information (9). These three dimensions are particularly salient for nursing students. In this context, insufficient readiness manifests as low professional identity and self-doubt, often linked to the “impostor phenomenon” and poor emotional regulation. Inadequate information is reflected in a limited awareness of diverse career pathways and an idealized view of the profession that clashes with clinical reality. Finally, conflicting information usually arises from external pressures, such as contradictory advice from mentors or the discrepancy between family expectations and the student’s personal career values. CDMD can negatively impact nursing students, causing them to delay choosing a career or leading them to make unsuitable choices. These consequences may weaken their professional identity and heighten the risk of job burnout and intentions to leave their jobs once they enter the workforce.

Career choice is a multidimensional and complex process, and is often a challenging decision-making process. Therefore, this study uses the Social Cognitive Career Theory (SCCT) and the Cognitive Information Processing Theory (CIP) as its theoretical framework to systematically understand and analyze the latent categories of CDMD and their influencing factors. From a broad perspective, SCCT emphasizes how individual characteristics, learning experiences, and environmental factors influence career decision-making behaviors by shaping self-efficacy, outcome expectations, and goal-setting (10).

Learning engagement (LE) refers to the positive psychological state students exhibit during the learning process, typically encompassing three dimensions: vigor, concentration, and commitment. Within the SCCT framework, LE is considered a crucial component of the learning experience. High levels of LE enable students to actively participate in professionally relevant activities and gain direct experience of their abilities and the nursing profession. This enhances their career decision-making self-efficacy and expectations of positive career outcomes, which facilitates their participation in career exploration and planning. LE has been positively correlated with undergraduate students’ learning gains (11), career maturity (12), and professional identity (13). Students with higher LE can cope with stress more effectively and use more positive coping strategies when facing career choices. This positive coping style helps reduce uncertainty in career decision-making, thus alleviating CDMD (14).

Perceived social support (PSS) reflects an individual’s subjective evaluation of their external support network, encompassing support from family, peers, and educational institutions (15). Within the SCCT framework, PSS is a key environmental factor. A PSS network can provide students with career information, emotional support, role models, and essential resources and opportunities. This support helps students cope more effectively with career uncertainties and challenges, thereby reducing decision-making difficulties (16). Studies have shown that college students ‘perceived social support can reduce the degree of CDMD by improving their psychological capital and career decision-making self-efficacy (17). Undergraduate nursing students with high social support are more likely to obtain career development information from family, school, or mentors, thereby reducing the difficulties associated with information gaps (18, 19).

**Figure 1:**
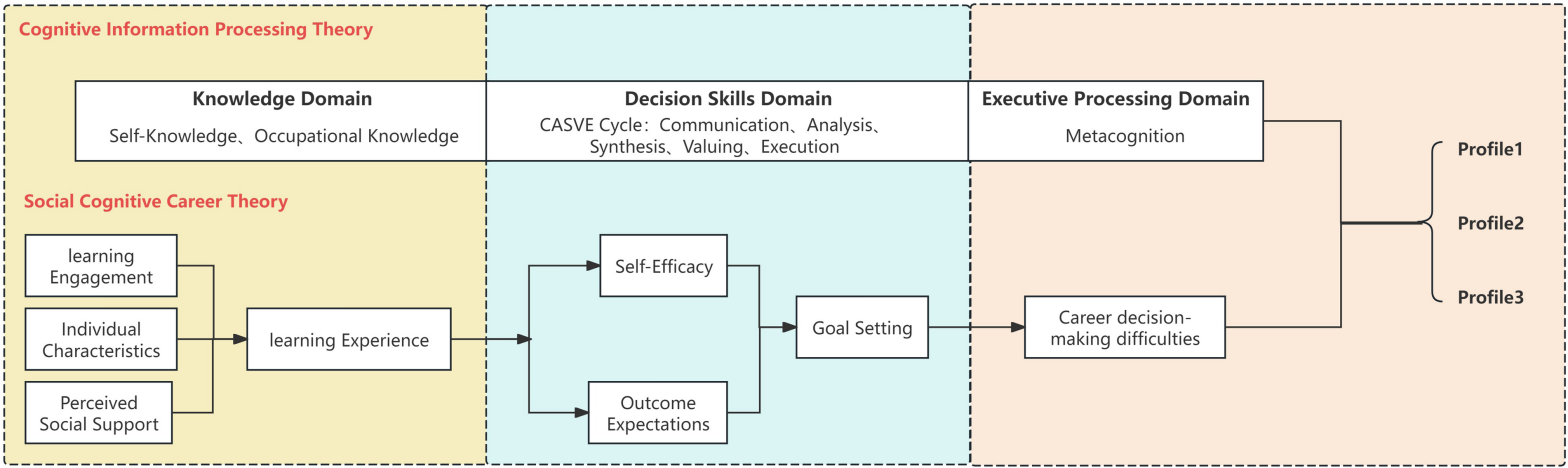
Theoretical model based on social cognitive career theory and cognitive information processing theory.

This model, as shown in Figure 1, illustrates how these constructs interact with learning experiences and contextual factors to influence career decision-making. SCCT provides a macro-level foundation to explore how demographic factors, PSS, and LE influence individuals’ engagement in career decision-making. Meanwhile, CIP theory offers a micro perspective, viewing career decision-making as a complex information-processing and problem-solving process. It focuses on individual differences at three levels of the information processing pyramid: the knowledge base (self-knowledge, vocational knowledge), decision-making skills (conceptualized as the CASVE cycle, a five-stage problem-solving process involving communication, analysis, synthesis, valuing, and execution), and metacognitive abilities (20). This approach helps analyze specific cognitive barriers or strengths at the micro level, explaining the diverse underlying characteristics of CDMD.

Most current research examines the overall impact of LE and PSS on university student’s career decision-making, often treating them as mediators. However, this variable-centered approach overlooks variability stemming from individual differences. Latent profile analysis (LPA) addresses this limitation by dividing the population into subgroups based on continuous variables, assessing the probability of subgroup membership for each individual, and presenting subgroup characteristics to aid in factor analysis (21). In summary, this study uses LPA, guided by SCCT and CIP, to identify the latent characteristics of CDMD among undergraduate nursing students and explores related influencing factors. This provides empirical evidence for developing more targeted career guidance and intervention strategies.

## 2 Methods

### 2.1 Design

A cross-sectional design was used in this study.

### 2.2 Setting and participants

A total of 562 nursing students were recruited using a convenience sampling method from three comprehensive universities and one teaching hospital in Zhejiang Province, China, between June and September 2024. Specifically, the sample consisted of 106 students from University A, 114 students from University B, 102 students from University C, and 240 students from the teaching hospital.

The inclusion criteria were: (1) full-time undergraduate nursing students enrolled in the first through fourth years of a four-year baccalaureate nursing program, and (2) those who provided informed consent and voluntarily agreed to participate. Participants were excluded if they were not enrolled due to reasons such as academic suspension or military service. The sample size was determined based on the requirements for latent profile analysis (LPA), which generally needs a minimum of 500 participants (22) to ensure robust model estimation and sufficient statistical power to detect meaningful latent categories. To account for a potential 10% sample loss, the target sample size was set at 550 participants.

### 2.3 Research tools

#### 2.3.1 Demographic questionnaire

The Demographic Questionnaire was developed by the research team based on a literature review (13, 23, 24). It includes items related to students’ age, year of study, gender, place of origin, only child status, parental education levels, family monthly income, class leadership role, whether nursing was their first-choice major, perceived excessive stress while studying nursing, and whether their parents work in healthcare.

#### 2.3.2 Multidimensional Scale of Perceived Social Support

This scale, initially developed by Dahlem et al. (25) and later revised and adapted into Chinese by Jiang (26), consists of 12 items that measure perceived support from family, friends, and significant others. Using a 7-point Likert scale, responses range from 1 (“strongly disagree”) to 7 (“strongly agree”). The total score is the sum of all items, ranging from 12 to 84. The higher the score, the higher the PSS. The Chinese version of the Multidimensional Scale of Perceived Social Support (MSPSS) has been validated in college students (Cronbach’s *α* = 0.92) (27). The Cronbach’s *α* coefficient for this scale in this study was 0.930.

#### 2.3.3 Utrecht Work Engagement Scale-Student

The Utrecht Work Engagement Scale-Student (UWES-S), developed by Schaufeli et al. (28) and adapted into Chinese by Fang et al. (29), was used to measure LE among undergraduate nursing students. This scale includes three dimensions: vigor, dedication, and absorption, with a total of 17 items. Responses are rated on a 7-point Likert scale, with scores ranging from 1 (never) to 7 (always). The total score ranges from 17 to 119. Higher scores indicate higher LE. The Cronbach’s *α* coefficient for this scale in the present study was 0.955.

#### 2.3.4 Career Decision-Making Difficulties Questionnaire

This scale was developed by Du (30) to assess CDMD among undergraduate students. The scale includes four dimensions: career information exploration, career self-exploration, career planning exploration, and career goal exploration, with a total of 16 items. Responses are rated on a 5-point Likert scale, ranging from 1 (strongly disagree) to 5 (strongly agree). Total scores range from 16 to 80. A higher score indicates a lower CDMD. The scale has been widely used among undergraduate nursing students (31, 32). The Cronbach’s *α* coefficient for this scale in the current study was 0.895.

### 2.4 Data collection

This study used a one-on-one guided electronic questionnaire survey method. Data were collected via the online platform www.wjx.cn. Data were collected by six trained surveyors who completed a 4-h standardized training session. The training covered the meaning of questionnaire items, standardized procedures, research objectives, effective communication with participants, maintaining neutrality, and handling potential issues that may arise. The following training, role-playing, and mock administrations assessed surveyor competency. Only surveyors who passed this assessment participated in formal data collection. Prior to formal data collection, a pre-survey was conducted with 30 representative undergraduate nursing students from participating institutions. Feedback from participants and surveyor observations led to refinements in questionnaire wording and adjustments to the online platform, enhancing clarity and usability. Pre-survey data were excluded from the final analysis. During formal data collection, questionnaires were distributed and collected through one-on-one guidance. For undergraduate nursing students in their freshman through junior years, surveys were administered during evening study sessions. For fourth-year students, data collection was conducted in clinical settings, as this academic year is primarily dedicated to mandatory, full-time clinical internships (during which they are referred to as “nursing interns”). With the assistance of clinical instructors, these students were surveyed during designated group meetings or their scheduled rest periods to avoid disruption to their practical training. In all settings, surveyors explained the study objectives, obtained informed consent, answered participants’ questions, and provided technical support for the online platform, all while maintaining neutrality to avoid influencing responses.

### 2.5 Quality control

To ensure consistency and minimize measurement error, the research team implemented several quality control measures. The online survey platform required that all mandatory fields be completed and restricted submissions to one valid response per device based on the IP address. Response times were monitored, and unusually short or long durations were flagged for review during the data cleaning process. The average completion time was approximately 7 to 10 min.

After data collection, a core team member exported the raw data to Excel 2024. Two independent team members conducted the initial data cleaning to exclude incomplete, logically inconsistent, or invalid responses. Out of 570 initially selected students, 562 valid responses were retained, resulting in a response rate of 98.60% after removing incomplete, duplicate, or invalid entries. Duplicate entries could arise if participants used multiple devices or networks, bypassing the initial IP address restriction. These were identified by screening for identical responses across key demographic variables and then manually confirmed by two researchers before removal.

### 2.6 Ethical considerations

Before the study began, the research team reached out to nursing department heads and hospital administrators to explain the study’s objectives, significance, and procedures. This helped secure institutional support and permission. All participants voluntarily signed an electronic informed consent form after receiving complete disclosure about the study’s purpose and methods.

Ethical guidelines were incorporated into the training for surveyors, focusing on maintaining neutrality, minimizing social desirability bias, and protecting participant confidentiality. Data privacy was further ensured through IP-based submission restrictions and secure storage on the survey platform. The study received approval from the Ethics Committee of Sir Run Run Shaw Hospital, Zhejiang University School of Medicine (Ethical Approval Number: 2024-0502).

### 2.7 Data analysis

LPA was conducted using Mplus 8.3, with the items from the CDMD serving as observed variables. The number of profiles was gradually increased. The optimal model was determined based on fit evaluation indices, and the resulting categories were labeled accordingly. The fit evaluation indices for the latent profile model included: (1) Information criteria, such as the Akaike information criterion (AIC), Bayesian information criterion (BIC), and adjusted Bayesian information criterion (aBIC), where smaller values indicate a better model fit; (2) Entropy, which ranges from 0 to 1, where values >0.8 indicate higher classification reliability; (3) The Lo–Mendell–Rubin adjusted likelihood ratio test (LMR) and the bootstrapped likelihood ratio test (BLRT), where *p* < 0.05 indicates that the model with *k* profiles fits better than the model with *k* − 1 profiles. Statistical analysis was performed using SPSS 27.0. Categorical data were described using frequencies and proportions, while continuous data with a normal distribution were presented as means ± standard deviations. To compare the latent categories of CDMD among undergraduate nursing students with different characteristics, chi-square tests, Fisher’s exact test, and one-way analysis of variance (ANOVA) were used. Unordered multinomial logistic regression was used to identify factors influencing the latent categories of CDMD among undergraduate nursing students. A *p*-value of less than 0.05 was considered statistically significant.

## 3 Results

### 3.1 Demographic characteristics of undergraduate nursing students

As shown in Supplementary Table 1, the study involved 562 undergraduate nursing students, consisting of 92 males (16.4%) and 470 females (83.6%), with an average age of 20.09 years (age range: 17–24 years). Among the participants, 59.8% held positions as class leaders, and 69.9% chose nursing as their first-choice program. Additionally, 60.5% reported experiencing significant stress during their studies. The distribution of participants by academic year was as follows: first-year students comprised 17.1% (*n* = 96), second-year students 16.2% (*n* = 91), third-year students 22.6% (*n* = 127), and fourth-year students 44.1% (*n* = 248). Participants were almost evenly distributed between urban (51.4%) and rural (48.6%) areas. More than half (56.6%) of the participants were not only children in their families. A significant portion of their mothers (48.6%) and fathers (46.8%) had completed at most a middle school education. Furthermore, the vast majority of participants’ parents were not employed in healthcare or nursing professions (92.5%). Regarding family monthly income, a majority of participants (73.3%) belonged to a broad middle-income bracket (5,000–20,000 RMB/month). The detailed distribution across five categories was: low (<5,000 RMB/month, 12.5%), low-middle (31.0%), middle (28.8%), middle-high (13.5%), and high (>20,000 RMB/month, 14.2%) (Supplementary Table 1).

### 3.2 Descriptive statistics of key variables

The descriptive statistics for PSS and LE are presented in Supplementary Table 1. The mean scores for PSS, LE, and CDMD were 64.16, 82.00, and 58.20, respectively.

### 3.3 Latent profile analysis of career decision-making difficulties among undergraduate nursing students

To explore the latent profiles of CDMD among undergraduate nursing students, a single-profile model was initially applied, with subsequent increases in the number of profiles to compare their fit indices. Table 1 presents the reference values of the model fit indices for LPA. Table 2 shows the fit indices of different latent class models when conducting LPA based on data from the Career Decision Difficulty Scale of 562 nursing undergraduate students. The *p*-value of the BLRT was significant for all models (<0.001), while the LMR yielded significant results for the two- and three-profile models. However, the *p*-value for the LMR in the four-profile model was not significant, leading to its exclusion from further analysis. The AIC, BIC, and aBIC values decreased progressively as the number of profiles increased. Additionally, Profile 3 showed a higher entropy value than Profile 2. Consequently, based on these indices, the three-profile model was identified as the optimal one.

**Table 1:** The model fit indices for LPA.

Indicators	Criteria
AIC	The smaller the value, the more accurate the classification of the model
BIC	The smaller the value, the more accurate the classification of the model
ABIC	The smaller the value, the more accurate the classification of the model
Entropy	Entropy >0.8 means 90% of individuals are accurately classified
BLRT	The *p*-value of <0.05 indicates that the class *k* model is superior to the class *k* – 1 model
VLMR-LRT	The *p*-value of <0.05 indicates that the class *k* model is superior to the class *k* – 1 model
Probability of class	Models with a probability of at least 5% for each category were classified more reasonably

AlC, Akaike information criteria; BIC, Bayesian information criteria; aBIC, adjusted Bayesian information criteria; BLRT, bootstrap likelihood ratio test; VLMR-LRT, Vuong–Lo–Mendell–Rubin likelihood ratio test.

**Table 2:** Fit indices of LPA for career decision-making difficulties profiles (*N* = 562).

Profiles	AIC	BIC	aBIC	Entropy	LMR	BLRT	Category probability
1	23548.304	23686.912	23585.328	/	/	/	/
2	21652.167	21864.411	21708.860	0.903	<0.001	<0.001	0.414/0.585
3	21185.484	21471.363	21261.846	0.911	0.026	<0.001	0.340/0.585/0.075
4	20799.366	21158.881	20895.397	0.923	0.142	<0.001	0.068/0.363/0.512/0.057

#### 3.3.1 Characteristics and naming of latent categories of career decision-making difficulties among undergraduate nursing students

**Figure 2:**
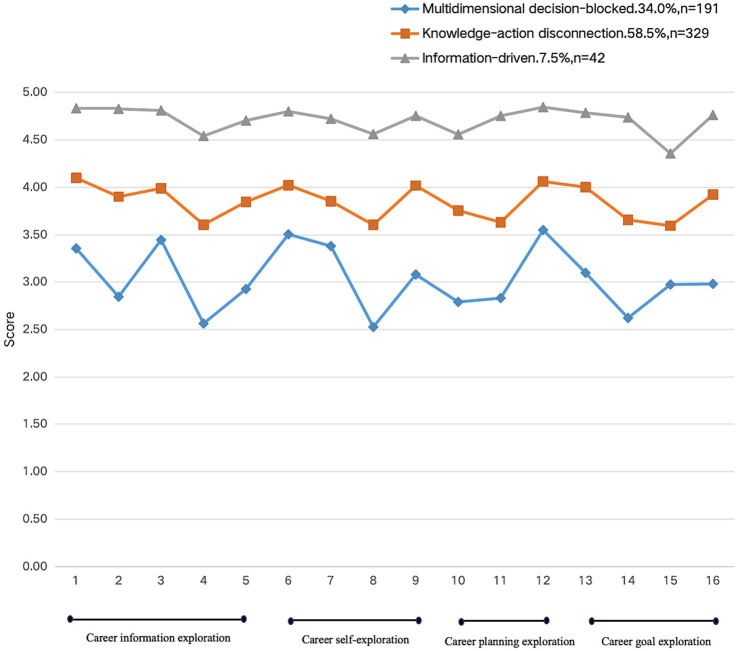
Three profiles of career decision-making difficulties among undergraduate nursing students based on LPA (*N* = 562).

Three distinct latent categories of CDMD among undergraduate nursing students were identified based on the scores from 16 observed variables, as shown in Figure 2. The 16 items on the scale can be categorized into four dimensions: Career Information Exploration (Items 1–5), Career Self-Exploration (Items 6–9), Career Planning Exploration (Items 10–12), and Career Goal Exploration (Items 13–16). The categories were named as follows:

Multidimensional Decision-Blocked group (191 participants, 34.0%): This group exhibited the lowest total score for CDMD, with consistently low scores across all four dimensions, indicating the highest level of CDMD.

Knowledge-Action Disconnection group (329 participants, 58.5%): This group scored above four on Items 1, 3, and 6, which are related to gathering career information and self-exploration. Moreover, their mean score in the Career Information Exploration dimension (3.89) was the highest among the four dimensions, indicating relatively strong abilities in career information and self-exploration. However, this group scored low on Items 4, 8, 11, and 15, which assess practical career actions, planning, and goal-setting. These low scores suggest limited practical engagement in career planning and a lack of clear goals or development plans.

Information-Driven Advantage group (42 participants, 7.5%): This group exhibited the highest total score on the CDMD scale, indicating the lowest level of CDMD. They had significantly higher scores on Items 1, 2, and 3, indicating excellent skills in gathering career information, particularly through the Internet.

#### 3.3.2 Between-group and dimension comparisons of latent profiles of career decision-making difficulties among undergraduate nursing students

To examine the heterogeneity between these profiles, the Kruskal–Wallis *H* test was used to compare scores on the career decision-making dimensions across the three groups, as the data were non-normally distributed. The results (Table 3) indicate significant differences in scores across the four dimensions of CDMD among the three groups (*p* < 0.001). *Post hoc* tests showed a gradual increase in scores for Career Information Exploration, Career Self-Exploration, Career Planning Exploration, and Career Goal Exploration from Profile 1 to Profile 3. Across all profiles, the Career Information Exploration dimension had the highest scores, while the Career Planning Exploration dimension had the lowest.

**Table 3:** Profiles differences in career decision-making difficulties and the results of *post hoc* analysis (*N* = 562).

Dimension	Profile 1	Profile 2	Profile 3	*H*	*p*	*Post hoc* analysis
Career Information Exploration	15.60 ± 2.587	19.80 ± 1.519	23.86 ± 1.475	442.577	<0.01	3 > 2 > 1
Career Self-Exploration	12.30 ± 2.042	15.13 ± 1.536	19.10 ± 1.340	331.310	<0.01	3 > 2 > 1
Career Planning Exploration	9.06 ± 0.128	11.26 ± 1.302	13.79 ± 1.406	230.587	<0.01	3 > 2 > 1
Career Goal Exploration	11.52 ± 0.161	15.43 ± 1.474	18.79 ± 1.554	435.804	<0.01	3 > 2 > 1

#### 3.3.3 Multivariate analysis of career decision-making difficulties among undergraduate nursing students

Univariate analysis (Supplementary Table 1) revealed significant differences in CDMD across the three categories based on gender, grade level, class leadership role, age, PSS, and LE (*p* < 0.05). These factors were then included in a multivariate analysis, with the three CDMD groups serving as the dependent variable. The “Multidimensional Decision-Blocked” group was used as the reference group. Gender (female as reference), grade level (fourth-year as reference), class leadership role (non-class leader as reference), age, PSS, and LE were included as independent variables in the unordered logistic regression analysis.

The results indicated that class leadership role, PSS, and LE were significant factors influencing CDMD among undergraduate nursing students. Compared to the “Multidimensional Decision-Blocked” group, undergraduate nursing students with a class leadership role were 2.981 times more likely to belong to the “Information-Driven Advantage” group. Furthermore, undergraduate nursing students with higher PSS and LE were more likely to be classified into either the “Information-Driven Advantage” group or the “Knowledge-Action Disconnection” group. Detailed results are presented in Tables 4, 5.

**Table 4:** Unordered logistic regression analysis results for the information-driven advantage group of career decision-making difficulties.

Variables	*B*	Standard error (SE)	Wald *χ*^2^ value	*p*	OR	95% CI
Age	0.106	0.282	0.141	0.708	1.112	0.640–1.932
Learning engagement	0.095	0.016	37.198	<0.001	1.099	1.066–1.134
Perceived social support	0.167	0.027	39.295	<0.001	1.182	1.122–1.245
Gender						
Male	0.692	0.383	3.273	0.070	1.998	0.944–4.229
Female (reference)						
Grade						
First-year	0.715	1.061	0.454	0.500	0.489	0.061–3.917
Second-year	0.629	0.813	0.599	0.439	1.876	0.381–9.232
Third-year	0.151	0.665	0.052	0.820	1.164	0.316–4.281
Fourth-year (reference)						
Serving as class leadership role						
Yes	1.092	0.432	6.404	0.011	2.981	1.279–6.946
No (reference)						

**Table 5:** Unordered logistic regression analysis results for the knowledge-action disconnection group of career decision-making difficulties.

Variables	*B*	Standard error (SE)	Wald *χ*^2^ value	*p*	OR	95% CI
Age	0.164	0.172	0.904	0.342	1.178	0.840–1.651
Learning engagement	0.056	0.008	54.578	<0.001	1.058	1.042–1.074
Perceived social support	0.053	0.011	21.816	<0.001	1.054	1.031–1.077
Gender						
Male	−0.374	0.247	2.282	0.131	0.688	0.424–1.118
Female (reference)	/	/	/	/	/	/
Grade						
First-year	0.365	0.600	0.370	0.543	0.694	0.214–2.250
Second-year	0.257	0.499	0.265	0.607	1.293	0.486–3.441
Third-year	0.128	0.328	0.152	0.696	0.880	0.462–1.674
Fourth-year (reference)	/	/	/	/	/	/
Serving as class leadership role						
Yes	0.351	0.224	2.453	0.117	1.420	0.916–2.202
No (reference)	/	/	/	/	/	/

## 4 Discussion

### 4.1 Latent profiles of career decision-making difficulties among undergraduate nursing students

This study is the first to use latent profile analysis LPA to identify potential profiles of CDMD among nursing students and their correlates. The results identified three distinct CDMD profiles among undergraduate nursing students: the “Multidimensional Decision-Blocked” group, the “Knowledge-Action Disconnection” group, and the “Information-Driven Advantage” group. Combining SCCT and CIP provides an explanatory framework for understanding CDMD among undergraduate nursing students.

Undergraduate nursing students in the “Multidimensional Decision-Blocked” group (34.0%) scored low across all four dimensions of CDMD, indicating significant challenges in their career decision-making process. From the SCCT perspective, the CDMD experienced by this group may be due to lower career self-efficacy (33) and inadequate social support (34). According to the CIP framework, this situation can be understood as a multi-level dysfunction within the decision-making “pyramid.” These students may lack essential self-knowledge and career information, which hampers their ability to effectively navigate the CASVE cycle—communicate, analyze, synthesize, evaluate, and execute. Consequently, they struggle to identify problems, gather and process information, generate options, and take action. Additionally, they may have underdeveloped metacognitive skills, such as managing decision-related anxiety and sustaining motivation, which further complicates their decision-making difficulties (35).

The majority of undergraduate nursing students (58.5%) belong to the “Knowledge-Action Disconnection” group, which indicates a disconnect in their career planning and goal-setting. This issue may stem from the complexity of their circumstances, which can hinder effective career decision-making, as well as a lack of essential skills. Research indicates that factors such as family disagreements, societal biases, and economic conditions can significantly impact students’ career choices (35, 36). The diversity and complexity of this information may confuse students, impacting their ability to establish clear career goals (37). Within the CIP framework, students in this group demonstrate practical skills in gathering information. However, they face challenges with the decision-making skills component of the CASVE cycle, particularly in analyzing, synthesizing, and evaluating complex information. This difficulty impedes their ability to translate knowledge into action during the career decision-making process (35).

Only 7.5% of undergraduate nursing students were classified as part of the “Information-Driven Advantage” group. This group scored significantly higher than the others in dimensions such as Career Information Exploration, Career Self-Exploration, Career Planning Exploration, and Career Goal Exploration. Members of this group excel at analyzing and integrating both internal and external information, enabling them to formulate clear career plans and goals that are tailored to their unique characteristics.

### 4.2 Factors associated with different career decision-making profiles among undergraduate nursing students

#### 4.2.1 Perceived social support

This study found that undergraduate nursing students who reported higher levels of PSS were more likely to be categorized into either the “Information-Driven Advantage” group or the “Knowledge-Action Disconnection” group. Furthermore, the positive predictive effect of social support on the “Information-Driven Advantage” group (OR = 1.182) was significantly more substantial than the effect on the “Knowledge-Action Disconnection” group (OR = 1.054).

This indicates that PSS significantly helps alleviate CDMD among undergraduate nursing students. This finding aligns with the SCCT, which highlights the importance of external environmental factors in the career decision-making process (48). Previous research has shown that social support can diminish CDMD by boosting career decision-making self-efficacy and psychological capital (17). Furthermore, positive coping strategies may mediate the relationship between PSS and CDMD (38). Individuals with higher levels of PSS tend to adopt a proactive attitude and engage in constructive actions when facing challenges. This proactive approach helps to minimize adverse effects, such as inconsistencies in information, during career exploration, which could otherwise obstruct effective decision-making. Conversely, individuals with lower PSS may not recognize the available help or resources around them, leading to passive coping behaviors (39). As a result, students with higher PSS are more likely to belong to the “Information-Driven Advantage” and “Knowledge-Action Disconnection” groups.

Additionally, this study found that the positive predictive effect of social support was significantly more substantial for the “Information-Driven Advantage” group than for the “Knowledge-Action Disconnection” group. This may be because students in the “Information-Driven Advantage” group are more adept at converting information and emotional support from teachers, parents, and peers into resources beneficial for career decision-making. In contrast, the “Knowledge-Action Disconnection” group faces obstacles in converting cognitive and behavioral processes into action. While emotional support may alleviate the stress associated with career decision-making, a lack of practical guidance within social support may lead to a “cognitive-behavioral disconnection” (40), thereby weakening its effect on this group.

#### 4.2.2 Learning engagement

This study found that undergraduate nursing students with higher LE were more likely to belong to the “Information-Driven Advantage” group (OR = 1.099) or the “Knowledge-Action Disconnection” group (OR = 1.058). According to SCCT, LE is considered a direct behavioral manifestation of the learning experience. Undergraduate nursing students with high LE, through active participation in professional activities, are more likely to develop a positive “outcome expectation” about their nursing career—that is, they believe the profession can fulfill personal values and offer growth opportunities (12). This sense of professional purpose and identity enhances career exploration motivation and clarifies career goals, ultimately alleviating CDMD (41). Within the CIP framework, high LE enriches the individual’s knowledge base in the first layer of the career decision-making “pyramid,” providing an informational foundation for subsequent stages of the CASVE cycle (communication, analysis, synthesis, evaluation, and execution). In addition, existing research suggests that students with bipolar (high/low) characteristics of career decision self-efficacy exhibit higher levels of academic engagement (42). This suggests a potential nonlinear relationship between LE, career decision self-efficacy, and CDMD, which can be further explored in future studies examining the type of career decision.

#### 4.2.3 Class leadership role

This study found that undergraduate nursing students who serve as class leaders are more likely to belong to the “Information-Driven Advantage” group (OR = 2.981). From an SCCT perspective, serving as a class leader represents a significant learning experience and an opportunity for environmental interaction. The class leadership role involves frequent interactions with teachers and classmates, organizing activities, and managing class affairs. These experiences offer individuals growth opportunities (43). Previous research indicates that class leaders in China generally exhibit higher academic performance, interpersonal skills, psychological resilience, social support, and subjective well-being (43). These factors contribute to enhanced confidence and decisiveness in career decision-making (44). Within the CIP framework, the class leadership experience directly impacts development in the decision-making and execution domains (metacognition). In performing these duties, class leaders engage in frequent communication and coordination with peers and teachers, helping them improve their ability to gather, process, and integrate information. This process fosters higher career maturity and professional self-concept (45), ultimately reducing their CDMD.

### 4.3 Limitations and future directions

This study has several limitations that warrant future research. First, the sample was limited to three comprehensive universities and one teaching hospital in Zhejiang Province, which may have reduced the generalizability and representativeness of the findings. Additionally, the distribution of students across academic years was uneven, reflecting variations in institution and hospital sizes. Future studies should broaden the sample to include participants from various regions for a more comprehensive understanding of CDMD among undergraduate nursing students. Additionally, the cross-sectional design limits the ability to establish causal relationships between the variables.

Furthermore, characteristics related to different profiles may change over time; therefore, longitudinal studies are needed to explore trends in CDMD. This study also revealed complex relationships between LE, PSS, and the identified CDMD profiles. Future research should incorporate additional relevant variables to explore these relationships further. Despite these limitations, this study employed LPA to identify distinct CDMD profiles and their associated characteristics, offering valuable insights for developing targeted interventions for specific subgroups of undergraduate nursing students.

### 4.4 Implications for education and practice

Nursing educators should dynamically assess the characteristics of nursing students facing CDMD and provide personalized support tailored to their profiles. For students experiencing “Multidimensional Decision-Blocked” difficulties, educators should proactively implement systematic career awareness education to help them understand career development pathways within nursing. For the majority of students in the “Knowledge-Action Disconnection” group, it is crucial to enhance their ability to integrate and process information.

This can be achieved through interactive teaching methods, such as career planning workshops (35) or role-playing activities (46), which help students translate theoretical knowledge into practical action. Additionally, educational institutions, such as schools and hospitals, should strive to create a positive learning environment that motivates active learning and encourages students’ investment in their learning (47). In addition to strengthening academic support, it is important to promote peer support, integrate family and community resources, and establish a multi-layered social support system (48).

## 5 Conclusion

This study identified three heterogeneous profiles of CDMD among nursing students: a majority “Knowledge-Action Disconnection” group (58.5%), a “Multidimensional Decision-Blocked” group (34.0%), and a small “Information-Driven Advantage” group (7.5%). Learning engagement and perceived social support were identified as significant protective factors, and most notably, holding a class leadership role emerged as the most powerful predictor for membership in the advantage group, increasing the odds nearly threefold. These findings advocate a transition from a uniform career support model to a profile-based approach. Customized interventions, aimed at addressing deficiencies such as the knowledge-action gap, are essential for more effectively preparing the future nursing workforce.

## Data Availability

The raw data supporting the conclusions of this article will be made available by the authors, without undue reservation.
