# Supplementary material for: Factors associated with career decision-making difficulties among undergraduate nursing students: a latent profile analysis
Source: Front Med (Lausanne). 2026 Jan 6;12:1644508. doi: 10.3389/fmed.2025.1644508 (PMC12816191; doi:10.3389/fmed.2025.1644508)
Supplement: Supplementary file 1 [file Table_1.docx]

**Supplementary Table 1.** **Demographic characteristics and the main independent variables description according to different** **career decision-making difficulties profiles (N=562).**

| **Variables** | **Categories** | **Multidimensional Decision-Blocked Group (34%)** | **Knowledge-Action Disconnection Group (58.5%)** | **Information-Driven Advantage Group (7.5%)** | **H/F** | **P-value** |
| --- | --- | --- | --- | --- | --- | --- |
| **Gender** | Male | 35 (18.3) | 44 (13.4) | 13 (31.0) | 9.214 | 0.001 |
|  | Female | 156 (81.7) | 285 (86.6) | 29 (69.0) |  |  |
| **Grade** | First-year | 43 (22.5) | 46 (14.0) | 7 (16.7) | 15.633 | 0.016 |
|  | Second-year | 26 (13.6) | 55 (16.7) | 10 (23.8) |  |  |
|  | Third-year | 53 (27.7) | 67 (20.4) | 7 (16.7) |  |  |
|  | Fourth-year | 69 (36.1) | 161 (48.9) | 18 (42.9) |  |  |
| **Serving as class leadership role** | Yes | 58 (30.4) | 142 (43.2) | 26 (61.9) | 17.112 | <0.001 |
|  | No | 133 (69.6) | 187 (56.8) | 16 (38.1) |  |  |
| **Enrolled as First Choice Major** | Yes | 128 (67.0) | 231 (70.2) | 34 (81.0) | 3.211 | 0.201 |
|  | No | 63 (33.0) | 98 (29.8) | 8 (19.0) |  |  |
| **Felt Excessive Stress in Nursing Studies** | Yes | 122 (63.9) | 197 (59.9) | 21 (50.0) | 2.901 | 0.234 |
|  | No | 69 (36.1) | 132 (40.1) | 21 (50.0) |  |  |
| **Place of Origin** | Urban | 100 (52.4) | 163 (49.5) | 26 (61.9) | 3.016 | 0.555 |
|  | Rural | 91 (47.6) | 166 (50.2) | 16 (38.1) |  |  |
| **Only Child Status** | Yes | 81 (42.4) | 142 (43.2) | 21 (50.0) | 0.829 | 0.661 |
|  | No | 110 (57.6) | 187 (56.8) | 21 (50.0) |  |  |
| **Mother’s Education Level** | Junior High or Below | 99 (51.8) | 160 (48.6) | 14 (33.3) | 6.463 | 0.167 |
|  | High School | 38 (19.9) | 84 (25.5) | 14 (33.3) |  |  |
|  | College or Above | 54 (28.3) | 85 (25.8) | 14 (33.3) |  |  |
| **Father’s Education Level** | Junior High or Below | 98 (51.3) | 149 (45.3) | 16 (38.1) | 3.192 | 0.526 |
|  | High School | 45 (23.6) | 87 (26.4) | 12 (28.6) |  |  |
|  | College or Above | 48 (25.1) | 93 (28.3) | 14 (33.3) |  |  |
| **Parents in Healthcare Industry** | Yes | 16 (8.4) | 20 (6.1) | 6 (14.3) | 4.649 | 0.325 |
|  | No | 175 (91.6) | 309 (93.6) | 36 (85.7) |  |  |
| **Family Monthly Income** **(RMB)** | Low  (<5000) | 30 (15.7) | 34 (10.3) | 6 (14.3) | 7.095 | 0.526 |
|  | Low–middle (5000-10000) | 56 (29.3) | 107 (32.5) | 11 (26.2) |  |  |
|  | Middle  (10000-15000) | 59 (30.9) | 92 (28) | 11 (26.2) |  |  |
|  | Middle–high (15000-20000) | 24 (12.6) | 47 (14.3) | 5 (11.9) |  |  |
|  | High  (>20000) | 22 (11.5) | 49 (14.9) | 9 (21.4) |  |  |
| **Age (years)**  **(Mean ± SD)** |  | 19.86±1.32 | 20.23±1.31 | 20.02±1.44 | 9.822 | 0.007 |
| **Perceived Social Support**  **(Mean ± SD)** |  | 57.32±11.06 | 64.16±8.99 | 74.45±8.71 | 63.580 | <0.001 |
| **Learning Engagement (Mean ± SD)** |  | 66.85±17.42 | 82±13.71 | 94.29±20.28 | 82.345 | <0.001 |
| **Career Decision-making Difficulties**  **(Mean ± SD)** |  | 48.48±5.58 | 61.63±3.64 | 75.52±3.90 | 886.501 | <0.001 |
